# Assessment of voluntary counseling and testing service utilization and associated factors among Debre Markos University Students, North West Ethiopia: a cross-sectional survey in 2011

**DOI:** 10.1186/1471-2458-13-243

**Published:** 2013-03-19

**Authors:** Girmay Tsegay, Melkie Edris, Solomon Meseret

**Affiliations:** 1 School of public Health, College of Medicine and Health Sciences, Debre Markos University, Debre Markos, Ethiopia; 2 School of Public Health, College of Medicine and Health Sciences, University of Gondar, Gondar, Ethiopia

**Keywords:** University students, VCT, Utilization, Associated factors

## Abstract

**Background:**

Voluntary Counseling and Testing (VCT) is the key entry point to prevention, care, treatment and support services, where people learn whether they are infected or not and to understand the implications of their HIV status and make informed choices for the future.

**Methods:**

A cross sectional study design was done among 753 students drawn from selected departments in Debre Markos University, Ethiopia, using multi-stage sampling technique. Self-administered questionnaire was used to estimate the prevalence of VCT service utilization and to assess associated factors. Data were entered and analyzed using SPSS version 16.

**Results:**

711 students were participated in the study, of whom, 521 were males. Majority (93.8%) were within the age range of 15 to 24 years. The mean age was 21.5 (3.2±); most of the respondents (91.6%) were single. Majority (81.4%) heard about the confidential VCT service, and their major sources of information were mass media (73.3%) and health workers (71.1%). The study revealed that 58.5% of the study participants had undergone voluntary counseling and testing. It was shown that VCT service utilization was significantly associated with availability of ART drug in VCT site, heard presence of confidentiality, perceived stigma, risk perception and knowledge about HIV.

**Conclusion:**

The major factors identified for increased VCT service utilization were knowledge about availability of ART drug in VCT site, information about confidentiality, absence of perceived stigma, higher risk perception and knowledge about HIV. Therefore, actions targeting on these predictors are necessary to effectively enhance the use of the VCT services utilization.

## Background

HIV/AIDS remains a major public health problem all over the world. UNAIDS (2008), estimate that 33 million people are living with HIV/AIDS globally. Sub-Sahara accounts for two-thirds of the global HIV/AIDS burden; Ethiopia is one of the Sub-Saharan countries highly affected by HIV/AIDS pandemic. According to the 2007 Ethiopian Ministry of Health report, the adult prevalence of HIV infection in Ethiopia was estimated at 2.1% [1-4].

Youth (15–24 years) are vulnerable to HIV because of the strong influence of peer pressure and the development of their sexual and social identities which often lead to experimentation [5]. The majority of young people in the age group (15–24 years) are at risk of HIV infection due to their engagement in unsafe sex, injection drug use, exposure to contaminated blood and blood products or unsterilized skin-piercing procedures [6]. The young age group is the most productive segments of the population that form the basic education sector which is vital to the creation of human capital this will affect both the demand and supply of education[7,8].

It has been estimated that most of the 37 million people worldwide living with HIV do not know that they are carrying the virus and the proportion is higher in countries worthily affected by the epidemic [9]. The first pillar of HIV prevention is voluntary counseling and testing (VCT). Despite the high number of people already living with HIV/AIDS, it is estimated that less than 10% are aware they are infected, mainly because of the limited availability, access, and use of VCT [10]. This fact greatly hinders efforts to respond to the AIDS epidemic, as people have to know if they are infected in order to access services [10].

Voluntary counseling and testing is also an effective strategy for facilitating behavior change for both clients, whose test result is either negative or positive [11]. Different studies have shown the effects of VCT including a decrease in unprotected sexual intercourse, a reduction in multiple partners, an increase in condom use, and more clients choosing abstinence. In addition, VCT is an important entry point to other HIV/AIDS prevention services, including prevention of mother to child transmission (PMTCT), prevention and management of HIV related illnesses, and social support (11).

Despite the high levels benefits of voluntary counseling and testing service, VCT utilization was low, particularly among young and active segment of populations. Research done in Uganda, Zambia, and among health care professional students of Kilimanjaro region revealed that voluntary counseling and testing service utilization were 10%, 14% and 34.5% respectively [12-14].

Various research findings in Ethiopia revealed that utilization of voluntary counseling and testing service is low and its level of utilization varies among different segments of the population [15-17]. Researches, conducted among university students corroborate this fact. For instance, a study done among Bahir Dar University students showed the utilization of VCT was 38.6%. Similarly, among Debre Birhan Teachers Training College students 35.19% of the respondents have ever been tested for HIV despite higher level of knowledge and favorable attitude towards VCT among the study population [15-17].

The aim of this study was to assess VCT service utilization and associated factors among Debre Markos University students, North West Ethiopia in 2011. The findings of the study will be helpful to expand and improve the service of VCT, contributing to the HIV/AIDS prevention and control programs in the University.

## Methods

This study was carried out in March 2011 among Debre Markos University students, Debre Markos Town, Ethiopia. Debre Markos is the capital city of East Gojjam Zone and is located in the North West part of the country bounded by Gozamen Woreda (district) in the North, South, and East, and Aneded Woreda (district) in the West. It covers an area of 6 million square meters and located on the main road of Addis Ababa (national capital)-Bahir Dar (regional capital), 300 km away from Addis and 265 km from Bahir Dar. It was estimated that the total population living was 70,857 people in 2008, of which about 21,257 were youth group. Two NGO clinics/FGAE and Marie Stops International providing reproductive health services to the target adolescent and youth groups; three government health center and one referral hospital provide VCT service. The only VCT centre in Debre Markos University campus is the one run by an NGO, African Initiative. Debre Markos University is one of the new universities in the country that were established in 2005. At the present time the university has seven faculties, Agriculture & Natural Resource, Business & Economics, Health Science, Law, Natural & Computational Sc., Social Sc. & Humanities, and Technology. The total number of regular students in all batches was 5746, out of which 1618 were female students.

This study utilized an institution-based cross-sectional study design with quantitative data collection method. The study population included all undergraduate students (Year I to year III) attending professional training at Debre Markos University. Those aged below 18 years and others who didn’t want to participate in the study and students who were seriously ill were excluded from the study.

The sample size was determined by Epi Info 2002 software package using a single proportion formula for cross-sectional survey, based on the prevalence of VCT service utilization (38.6%) during the past year among Bahir Dar University students [17]. Using 5% margin of error at 95% confidence level, the sample size required was 753 after considering 10% non-response rate. Multi-stage sampling technique was used to select study subjects. Firstly, students in the University were stratified by their field of study as health science and non health science. Secondly, one department from health science and five departments from non health science were selected using simple random sampling based on proportion. Thirdly, students in each field of study were further stratified by their year of study assuming that their field of study and duration of stay in the campus affect their VCT utilization. Finally, students were selected from each batch proportionally by simple random sampling technique using computer generated random numbers.

Data collection was done by pre-tested, pre-coded, and self- administered questionnaire with open and closed ended questions. This structured questionnaire adapted from similar previous studies and sample of questionnaire that was modified to the study setting. The questionnaires were self-administered to collect socio-demographic information and other important variables that include: VCT utilization, individual’s knowledge, sources of information and other variables. Pretesting of the questionnaire was performed on few students of Bahir Dar University students to verify clarity of the instrument used.

The questionnaire is originally prepared in English language and then translated to Amharic and again re-translated to English by language experts for consistency. Data collectors were nurses that were recruited and trained for one day by the principal investigator. Each questionnaire filled was checked for completeness of the information jointly by the facilitators. To reduce the error arising from respondents, 5% of randomly selected questionnaire was rechecked for consistencies by the principal investigator.

The collected data were cleaned, coded, entered into SPSS and analyzed using SPSS computer soft-ware package version 16. Summary statistic of socio-demographic variables was resented using frequency tables and graphs. Bivariate analysis was done and variables with p-value less than 0.2 were included in the multiple logistic regression analysis which was performed to assess the association between VCT utilization and various explanatory variables. P-value less than or equal to 0.05 was taken as cut of value to be significant. Odds ratio and 95% confidence interval was also constructed along with the corresponding p value.

The ethical review committee of the School of Public Health in Gonder University, College of Medicine and Health Sciences, approved the study for its ethical and scientific merit.

Communication was held with Debre Markos University and the University supported the undertakings of this study in writing to all the respective sampled departments. Informed verbal consent was also obtained from the respective students for their participation after the nature of the study was fully explained in their local languages. The right to withdraw from the study at any time was also communicated and respected. At the end of the interview all students were advised and encouraged to follow their peer groups to utilize the VCT service.

## Results

From the total of 753 students, 711 completed the questionnaire adequately making the response rate 94.4%. The mean age of the students was 21.5 with standard deviation of 3.2. The number of students from rural areas encompasses 406 (57.1%). About 521 (73.3%) and 651 (91.6%) of students were males and single respectively. Around 667 (93.8%) of students were found at the age group of 15–24, and 636 (89.5%) were Orthodox Christian religion followers. Five hundred ninety five (83.7%) students belong to Amhara ethnic group and 588 (82.7%) were non-health science students (Table 1).

**Table 1:** Socio demographic characteristics of Debre Markos University students, March 2011

**Socio-demographic characteristics**	**Frequency**	**Percentage (%)**
**Residence**		
Urban	305	42.9
Rural	406	57.1
**Sex**		
Male	521	73.3
Female	190	26.7
**Marital status**		
Single	651	91.6
Married	52	7.3
Divorced/separated	8	1.1
**Age category**		
15-24	667	93.8
25-34	27	3.8
35-44	17	2.4
**Educational status**		
First year	279	39.3
Second year	237	33.3
Third year	195	27.4
**Religion**		
Orthodox	636	89.5
Muslim	37	5.20
Protestant	35	4.90
Others	3	0.40

About 579 (81.4%) of students have heard the presence of confidential VCT service and 668 (73.3%) of them were heard from mass media as a primary source of information. The major reason for utilization of voluntary counseling and testing by students were to know their status 341 (82%) followed by for marriage 29 (7%). Six hundred eleven (85.9%) of students know the availability of ART drug in the voluntary counseling and testing sites. Around 561 (78.9%) students prefer face to face way to get HIV/AIDS test result and about 682 (96%) students were knowledgeable about HIV/AIDS (Table 2).

**Table 2:** Knowledge, source of information about VCT service utilization and other characteristic of students within the last 12 months, Debre Markos University, March 2011

**Variables**	**Frequency**	**Percentage (%)**
**Perceived confidentiality of VCT**		
Yes	579	81.4
No	132	18.6
**Source of information**		
Mass-media	668	73.3
Health workers	510	71.7
**Knowledge about availability of ART in the VCT site**		
Yes	607	85.4
No	104	14.6
**Perceived stigma after Positive result**		
Yes	251	35.3
No	460	64.7
**Ever had Sex**		
Yes	209	29.4
No	502	70.6
**Preferable ways of getting HIV test result**		
Face to face	561	78.9
Secret letter	118	16.6
others	32	4.5
**VCT utilization**		
Yes	416	58.5
No	295	41.5
**HIV Risk perception**		
Yes	473	66.5
No	238	33.5
**Age at first sex**		
10-14	3	1.4
15-19	104	50
20-24	89	42.8
25-30	12	5.8
**Knowledge about HIV/AIDS**		
Knowledgeable	682	96
Not knowledgeable	29	4
**Willingness to VCT**		
Yes	626	88
No	85	12
**Perceived Importance of VCT**		
Yes	691	97.2
No	20	2.8

**Figure 1:**
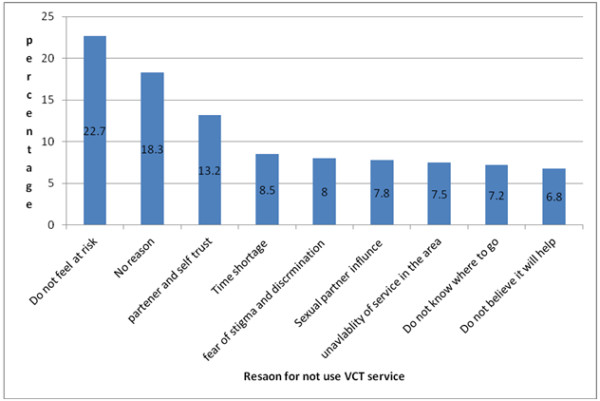
Bar chart showing reason given by the students for not using voluntary Counseling and testing service within the last 12 months, Debre Markos University, March 2011.

Study participants were compared for reported reasons of not utilizing voluntary counseling and testing (VCT) service for HIV/AIDS, about 67 (22.7%) were reported unwilling to utilize VCT service due to didn’t feel at risk, 54 (18.3) have no reason for not utilizing VCT, 39(13.2%) of the students were trusting themselves and their partners (Figure 1).

Fear of stigma and discrimination associated with positive test HIV/AIDS result was reported by 24 (8%) of students. Students who reported unavailability of the service nearby and considering the service is not useful were account for 22 (9.1) and 20 (8.3) respectively (Table 3, Figure 1).

**Table 3:** Associations of various characteristics of students by VCT service utilization within the last 12 months, Debre Markos University, March 2011

**Variables**	**VCT utilization**		**COR (95% CI)**	**AOR (95% CI)**	**P-value**
	**Yes (n = 416)**	**No (n = 295)**			
**Knowledge of HIV**					
Knowledgeable	407	275	3.3 (1.47, 7.33)	3.69 (1.19, 11.49)	0.04
Not knowledgeable	9	20	1	1	
**Confidentiality**					
Yes	357	222	1.99 (1.36, 2.92)	3.00 (1.65, 5.49)	0.001
Non	59	73	1	1	
**Perceived risk**					
Yes	351	111	8.95 (6.28, 12.70)	2.40 (1.37, 4.23)	0.002
No	65	184	1	1	
**Availability of ART**					
Yes	375	232	2.48 (1.62, 3.80)	3.11 (1.62, 5.99)	0.001
No	41	63	1	1	
**Perceived stigma**					
Yes	16	235	0.01 (0.006, 0.018)	0.013 (0.007, 0.024)	0.01
No	400	60	1	1	

## Discussion

Voluntary Counseling and Testing (VCT) is proven to be one of the most powerful weapons in halting the spread of HIV/AIDS. It is known to be a very important component of HIV/AIDS prevention strategies. Despite the fact that various studies have shown low utilization of VCT service particularly in developing countries this study tried to look in to the level and associated factors of VCT utilization by students in Debre Markos University (14).

The finding indicates that Knowledge of modes of HIV/AIDS transmission and prevention among the students was high. 96% of the respondents were knowledgeable of HIV/AIDS transmission and prevention.

The result also revealed on sexual practice and perceived risk of becoming infected with HIV among the students. Of all the respondents 29.4% had sexual experience, of which, 50% started sex at the age of 15–19 years. The respondents that felt being at risk of HIV infection comprised 66.5% of all.

In this study 81.4% of respondents heard about the availability of confidential VCT services and their major primary sources identified were mass-media and health workers.

The overall prevalence of VCT utilization in the past12 months was 416 (58.5%) with slightly higher utilization by females as compared to males. This result is higher when compared with the health care professional students of Kilimanjaro region revealed that voluntary counseling and testing utilization was 34.5% [12,13]. It is also higher than the study conducted among students in Bahir Dar University and Debre Birhan Teachers Teaching College with VCT utilization of 38.6 and 35.19 respectively (15,17).

The possible reasons for this could be due to recent accelerated expansion of the VCT service carried out through an increased advocacy and social mobilization in higher institution as well as country-wide and the study participant explained by the high risk perception and more knowledge regarding the VCT benefits.

The most commonly cited reason that VCT users gave for getting an HIV test was 82% to know their status followed by 7% for marriage. This is similar with finding in Debre Birhan Teachers Teaching College which showed 84.15% and 12.20% respectively [15]. The two main reasons mentioned by study participant for not using VCT service were not feeling at risk and trusting one-self and their sexual partner.

Overall, 88% study subjects showed their willingness to undertake HIV counseling and testing in the future. However, this high percentage of willingness to take the VCT service by the study participants different from the actual practice. This might be due to less mobilization activity and fear of testing and its consequences, other reasons to this may be due to lack of perceived benefits of VCT.

Knowledge about HIV/AIDS and VCT utilization has positive association. Students who have knowledge about HIV were 3.69 times more likely to utilize VCT service as compared to those who did not have knowledge about HIV. This finding is supported by the assumption that VCT users could have more exposure/information/knowledge regarding HIV/AIDS before they came to VCT centers (15). This again may indicate the information to be disseminated through health education and counseling sessions may benefit from the inclusion of such topics during the respective sessions, and continuous mass media activity.

In this study, socio-demographic characteristics of the students were not significantly associated with VCT service utilization, which is contradicted with the study conducted among students in Mekele University [16]. Possible reason could be recent accelerated expansion of the VCT service carried out through an increased advocacy targeting disparities in sex, region, religion, age and ethnicity etc.

This study identified the availability of ART as a positive predictor of VCT acceptance. Students who know the availability of ART in the VCT site were 3.12 times more likely to utilize VCT service as compared to those who didn’t know availability of ART in the VCT site. The provision of ART would have a significant effect in prolonging life and this would have an impact on the students in creating positive attitude and acceptance towards the service. Making every effort to make ART available would increase VCT acceptance by all students and students, who perceive risks associated with the HIV/AIDS test result were 2.4 times more likely to utilize VCT service as compared to their counterpart.

In this study perceived stigma associated with the positive test result was found to be important factor of VCT service utilization. Students who perceive stigma and discrimination were 0.013 times less to likely utilize VCT service as compared to their counterparts. This is in line with the study in Bahir Dar University students, where perceived stigma and discrimination was known to be strong predictor of voluntary counseling and testing [17].

The other finding of this study was the positive association of presence of confidentiality on the VCT site and utilization of VCT service among students. Students who heard the presence of confidentiality in the VCT service site were 3 times more likely to utilize VCT service as compared with those who didn’t hear the presence of confidentiality in the VCT service sites.

This study encountered a number of limitations, among which, as with any observational study, the possibility of residual confounding effect of some factors cannot be excluded. This may result in spurious associations of the factors with some events, and we thus guarded against this possibility by careful sequential building of models in our analyses. Another limitation of this study was findings from this study may not be generalized to the whole population of the young people because the study involved only those young people who are in higher institutions.

## Conclusion

Firstly, majority of the students knew the existence of confidential VCT services and large proportion of them utilized the service. Secondly, five important variables were identified as predictors for the increased VCT service utilization. The odds of having used VCT significantly increased with knowing availability of ART drug in VCT site, heard presence of confidentiality, absence of perceived stigma, higher risk perception, and knowledge about HIV. Therefore, actions targeting on those predictors are necessary to effectively enhance the use of the VCT services which allows students to know their HIV sero-status and prepare for treatment or care which could represent a reasonable commitment towards HIV/AIDS prevention in higher institutions.

## Competing interests

The authors declare that they have no competing interests.

## Authors’ contributions

GT: conception and initiation of the study, design, implementation, analysis and writing. ME: design, implementation of the study and co-writing. SM: design, implementation and co-writing. All authors read and approved the final manuscript.

## Authors’ information

Girmay (BSc, MPH) is lecturer at Debre Markos University, College of Medicine and Health Science, Debre Markos, Ethiopia.

Melkie (professor) is lecturer at Gondar University, college of Medicine and Health Science, Institution of Public Health, Gondar, Ethiopia.

Solomon (assistance professor) is lecturer at Gondar University, College of Medicine and Health science, Institution of Public Health, Gondar, Ethiopia.

## Pre-publication history

The pre-publication history for this paper can be accessed here:

http://www.biomedcentral.com/1471-2458/13/243/prepub
